# Data on player activity and characteristics in a Serious Game Environment

**DOI:** 10.1016/j.dib.2019.104965

**Published:** 2019-12-09

**Authors:** Sa Liu, Min Liu

**Affiliations:** a Harrisburg University of Science and Technology, United States; b University of Texas at Austin, United States

**Keywords:** Computer log data, Serious games, Player behavior, Player characteristics

## Abstract

This dataset presents 159 players' computer log data when they play a Serious Game along with their characteristics data including problem-solving performance scores, demographic information, metacognition, and goal orientation measurements. A total of 85,194 log files were recorded during one-hour game play. The data here are related to the research paper entitled “The Impact of Learner Metacognition and Goal Orientation on Problem-Solving in a Serious Game Environment” by Liu and Liu [1]. The data collected would provide insights on the relationship among individual player characteristics, in-game behavior and performance in the game.

**Table:** Specifications Table

Subject	Computer Science
Specific subject area	Human-Computer Interaction
Type of data	Table
How data were acquired	Computer log data in the Serious Game named Alien Rescue Survey Instruments
Data format	Raw Analyzed Filtered
Parameters for data collection	The data collection considered the following parameters, including 1) Participants Demographics–over 18 years old, have not played the game, and voluntary participation; 2) Sample size–G-power was used to determine sample size for conducting multiple regression analyses; 3) Environment impact–participants played the game in a laboratory controlled setting to reduce the effects of environment.
Description of data collection	In a laboratory setting, participants were given an online survey on their demographic information, metacognition and goal orientation before playing the Serious Game. After the survey, each participant played the game up to an hour.
Data source location	University of Texas at Austin Austin, Texas United States
Data accessibility	With the article
Related research article	Sa Liu, Min Liu The Impact of Learner Metacognition and Goal Orientation on Problem-Solving in a Serious Game Environment Computers in Human Behavior https://doi.org/10.1016/j.chb.2019.08.021

**Table:** 

**Value of the Data** • The data highlights computer log data and player individual characteristics, which could be used to understand the impact of individual player characteristics on their behavior pattern in game environments. • Researchers and designers can benefit from these data, as it provides insights on human-computer interaction in game environments, particularly based on individual players' characteristics. • The structure and analysis methods of the log data can be generalized to analyse player behavior in other game environments. It also provides a valuable reference for future data collection on computer log data. • The data can be further analyzed to investigate other player in-game behavior, such as decision making and problem-solving.

## 1 Data

The data presented in this article is associated with the published research paper entitled “The Impact of Learner Metacognition and Goal Orientation on Problem-Solving in a Serious Game Environment” [1]. Four csv files are included in this article: Log_Raw.csv, Duration_Charateristics.csv, Gates.csv, and Consoles.csv. Log_Raw.csv file has five columns to demonstrate computer log raw data, including player action (i.e., Click, Open, Close, Select, Add, Create, Edit, and Change), timestamp (i.e., the time that the player made the action, e.g., Wednesday, November 29, 2017 10:35 a.m.), tool (e.g., Alien Database, see Table 1 for all 10 tools provided in the environment), and player ID (assigned randomly by computer, e.g., 0eMO7JyKimSMtLXKqCv9c6q9PcI2). A total of 85,194 player actions were captured in the log data.

**Table 1:** Descriptions of cognitive tools provided in alien rescue.

Name	Tool Functions
Alien Database	Presents textual descriptions and 3D visuals of the alien information including alien home solar system, journey to Earth, and the characteristics and needs of each species.
Solar System Database	Provides information on the planets and moons in our solar system. Intentionally incomplete data ensures learner must design and send probes to test hypotheses.
Missions Database	Presents information on the mission, technology and findings of historical NASA probe launches.
Concepts Database	Provides supplemental instruction on scientific concepts presented elsewhere in AR.
Spectra	Provides students spectral information to interpret spectral data encountered in AR.
Periodic Table	Provides a periodic table of all the elements for reference.
Notebook	Provides a place for students to take notes as they engage in solving the central problem.
Probe Design Center	Allows students to design, build and send probes to gather data on worlds in our solar system.
Mission Control Center	Provides an interface to view data from launched probes.
Message Tool Problem Solution Form	Allows students to read messages regarding the background story. Provides the Solution Form, which allows students to submit their planets recommendations and rationale for review by teachers.

Duration_Charateristics.csv file contains filtered data for 159 players, including the player time spent duration on each of the 10 tools, Gender, player metacognition score (i.e., MC_Average, range from 0 to 100), game performance score (i.e., solution_score, range from 0 to 7), and six goal orientation scores (i.e., task-approach– TAP_ALL, task-avoidance– TAV_ALL, self-approach– SAP_ALL, self-avoidance– SAV_ALL, other-approach– OAP_ALL, and other-avoidance goal orientation–OAV_ALL, range from 0 to 21).

Gates.csv file presents raw data regarding player movement in the game environment. It contains four columns including player gate access (i.e., ProbeDoor1, AlienDoor1, and MissionDoor1), timestamps, and player ID. A total of 4460 gate access actions were recorded.

Consoles.csv file contains raw data on player actions regarding console control in the game environment. There are four columns in the file, including player actions (i.e., Open and Close), timestamps, console (i.e., Alien DB, Communication Centre and Probe Design) and player ID. A total of 5916 console actions were recorded.

## 2 Experimental design, materials, and methods

This data collection utilized Alien Rescue (AR, http://alienrescue.edb.utexas.edu) game environment [2]. In AR, a player needs to utilize the information provided within this environment to find suitable planets for six displaced alien species and explain their rationale at the end of the game. 159 undergraduate students were recruited from a large public university in the southwestern United States. All participants were over 18 years old, and had not played AR prior to participating in the study. All players voluntarily participated in the study.

Firstly, in a laboratory setting, participants were given an online survey on their demographic information, metacognition (Metacognitive Awareness Inventory, MAI) [3] and goal orientation (3 X 2 Achievement Goal Orientation Inventory) [4] before playing AR. After the survey, the researcher played the opening video scenario, which provided the context and described the problem. The participants were asked to find the home planet for one alien named Jakala-Tay. Then each participant was given a username and password to login to the AR environment and had 60 minutes to work independently on the problem. At the end of the playthrough, the participants were asked to submit home solutions for Jakala-Tay. Their solution scores and activity logs in AR were collected during the problem-solving processes.

K-means cluster analysis was used in SPSS to determine player metacognitive levels and goal orientation groups. Tableau and R were used to visualize player log data. These log data were grouped into two types of actions during problem-solving in the game including room visit action (sequences) and tool use action (frequency, duration). Chord Diagrams [5] and the R circlize package [6] were used to visualize player tool use frequency and duration of action. In addition, the data was further analyzed using similarity measure, which is a statistical function to quantify the (dis)similarity of two objects, such as text strings, documents, audio files, digital photographic images, DNA sequences, and other digitized objects for pattern recognition [7,8]. Specifically, three different similarity measures including *Cosine (Cos), Jaccard (Jac)*, and *Longest Common Substring (LCS)* coefficients were used [9] to compare room visit sequences of player groups based on their metacognition and goal orientation groups.
